# Letter from the Editor in Chief

**DOI:** 10.19102/icrm.2019.100108

**Published:** 2019-01-15

**Authors:** Moussa Mansour


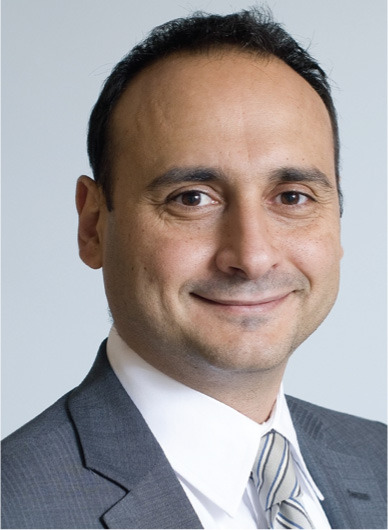


Dear Readers,

The number of patients with cardiac implantable electronic devices (CIEDs) is expanding. The resulting amount of work needed to manage the increasing number of CIEDs in existence as well as to analyze the collected information is staggering and is placing a significant strain on cardiac arrhythmia services in many hospitals. Requests to change device settings in both inpatient and outpatient locations are becoming more common, and the existence of large backlogs of unchecked reports is not unheard of. As a result, device clinics are hiring more and more technicians, nurses, and physicians in an effort to keep up with the growing demand, with varying degrees of success. In the long-term, satisfying the needs of this current trend with existing resources is not sustainable. Creative automated and remote solutions using advanced software technologies capable of handling large workloads are needed.

This issue of *The Journal of Innovations in Cardiac Rhythm Management* contains an interesting article aiming to tackle an aspect of this growing problem. It is titled “Remote Control of Cardiac Implantable Electronic Devices: Exploring the New Frontier—First Clinical Application of Real-time Remote-control Management of Cardiac Devices Before and After Magnetic Resonance Imaging.” In it, Kloosterman et al.^1^ describe an innovative solution for the management of patients with CIEDs undergoing magnetic resonance imaging (MRI) consisting of remote device management implemented before and after the imaging study. The task was successfully completed in 50 MRI sessions without adverse events, using encrypted communication lines. More importantly, the algorithm was overwhelmingly accepted by the patients involved.

It is true that the findings in this report are preliminary, and the study design has many limitations. However, the article is important because it highlights the issue introduced by the growing needs of CIED management and presents a resolution for one aspect of it. The described solution of remotely accessing and managing of devices can be modified for other applications and under different circumstances, including for supervising patients in the emergency department and in distant locations. Another aspect of the problem is the wrangling of large amounts of data received from device interrogations and ambulatory monitors. Artificial intelligence and machine learning in combination with seamless integration between CIEDs and electronic health records could represent a fully automated solution for use in many clinical scenarios, such as the detection of atrial fibrillation during routine checks, without human intervention.

Overall, achieving completely remote CIED management and fully automated data analysis requires not only the development of advanced technology but also a strong collaboration between device companies, electronic medical records developers, health care providers, hospitals, and regulatory agencies. While such a task may not be easily achievable at this time, it is of such importance that every effort should be made to complete it.

At this time, I would also like to take the opportunity to thank Dr. Asirvatham for his service to *The Journal of Innovations in Cardiac Rhythm Management* and welcome his replacement, Dr. Arash Aryana, MD, PhD, who will be taking over as the new Innovative Techniques section editor for the journal. Dr. Aryana is a board-certified cardiac electrophysiologist who received his training at the Massachusetts General Hospital/Harvard Medical School in Boston, MA. He serves as the Chair of the Department of Cardiology and Cardiovascular Surgery as well as the Medical Director of the Cardiovascular Service Line at the Mercy General Hospital/Dignity Health Heart and Vascular Institute in Sacramento, CA. His areas of expertise and research include catheter ablation of cardiac arrhythmias, with a particular interest in atrial fibrillation and ventricular tachycardia ablation. He is also the director of a nationally-recognized percutaneous epicardial ablation training program. To date, he has trained more than 100 practicing cardiac electrophysiologists and cardiac electrophysiology fellows throughout the country on this approach.

Best wishes for a fantastic and productive 2019.

Sincerely,


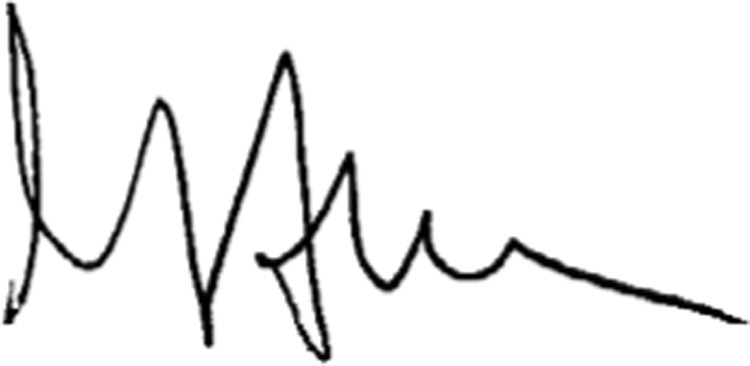


Moussa Mansour, md, fhrs, facc

Editor in Chief

The Journal of Innovations in Cardiac Rhythm Management

MMansour@InnovationsInCRM.com

Director, Atrial Fibrillation Program

Jeremy Ruskin and Dan Starks Endowed Chair in Cardiology

Massachusetts General Hospital

Boston, MA 02114
